# Supplementary material for: Impact of Weight on Clinical Outcomes of Edoxaban Therapy in Atrial Fibrillation Patients Included in the ETNA-AF-Europe Registry
Source: J Clin Med. 2021 Jun 29;10(13):2879. doi: 10.3390/jcm10132879 (PMC8269173; doi:10.3390/jcm10132879)
Supplement: Supplementary file 1 [file jcm-10-02879-s001.zip › jcm-1244438-supplementary.pdf]

Table S1. Baseline demographics and clinical characteristics of patients included in ETNA-AF-Europe according to additional body weight categories.

	ETNA-AF EU	Body Weight [kg]			
	Total	<50 kg	≥50 and ≤60 kg	>100 and ≤120 kg	>120 kg
Patients, N (%)	13,092 (100%)	152 (1.2%)	1158 (9.1%)	1159 (9.1%)	287 (2.3%)
Female, *n* (%)	5661 (43.2%)	139 (91.4%)	1006 (86.9%)	253 (21.8%)	70 (24.4%)
Age, years					
mean ± SD	73.6 ± 9.46	78.3 ± 9.71	78.5 ± 8.49	67.5 ± 9.43	62.7 ± 9.62
median (Q1; Q3)	75 (68; 80)	80 (72; 85)	79 (74; 84)	68 (62; 74)	63 (57; 69)
< 65 years	1994 (15.2%)	9 (5.9%)	60 (5.2%)	390 (33.7%)	151 (52.6%)
[65; 75)	4456 (34.0%)	35 (23.0%)	268 (23.1%)	496 (42.8%)	105 (36.6%)
[75; 85)	5268 (40.2%)	68 (44.7%)	550 (47.5%)	259 (22.4%)	30 (10.5%)
≥ 85 years	1372 (10.5%)	40 (26.3%)	280 (24.2%)	13 (1.1%)	1 (3.4%)
Body weight,kg (min,max)	(38.0, 193.0)	(38.0, 49.0)	(50.0, 60.0)	(10.0, 120.0)	(121.0, 193.0)
mean ± SD	81.0 ± 17.29	45.8 ± 2.88	56.2 ± 3.26	108.5 ± 5.60	135.0 ± 14.02
median (Q1; Q3)	80.0 (70.0; 90.0)	47.0 (44.0; 48.0)	57.0 (54.0; 56.0)	107.0 (104.0; 113.0)	130.0 (125.0; 140.0)
≤ 60 kg	1310 (10.3%)	152 (100.0%)	1158 (100.0%)	0 (0.0%)	0 (0.0%)
(60; 80]	5565 (43.9%)	0 (0.0%)	0 (0.0%)	0 (0.0%)	0 (0.0%)
(80; 100]	4346 (34.3%)	0 (0.0%)	0 (0.0%)	0 (0.0%)	0 (0.0%)
> 100 kg	0 (0.0%)	0 (0.0%)	0 (0.0%)	253 (100.0%)	287 (100.0%)
BMI, kg/m² (min, max)	(13.8, 68.6)	(13.8, 24.8)	(16.7, 28.5)	(24.0, 51.1)	(30.6, 68.6)
mean ± SD	28.1 ± 5.11	18.8 ±1.85	22.1 ± 2.10	35.1 ± 3.99	42.6 ± 5.84
median (Q1; Q3)	27.3 (24.7; 30.7)	18.7 (17.8; 19.7)	22.0 (20.7; 23.4)	34.5 (32.3; 37.2)	41.5 (38.6; 45.5)
<18.5 kg/m²	115 (0.9%)	67 (45.0%)	43 (3.8%)	0 (0.0%)	0 (0.0%)
[18.5; 25)	3341 (26.7%)	82 (55.0%)	984 (86.3%)	1 (0.1%)	0 (0.0%)
[25; 30)	5377 (42.9%)	0 (0.0%)	113 (9.9%)	81 (7.1%)	0 (0.0%)
[30; 35)	2544 (20.3%)	0 (0.0%)	0 (0.0%)	551 (48.3%)	15 (5.2%)
[35; 40)	792 (6.3%)	0 (0.0%)	0 (0.0%)	377 (33.0%)	87 (30.4%)
≥ 40 kg/m²	352 (2.8%)	0 (0.0%)	0 (0.0%)	132 (11.6%)	184 (64.3%)
≥ 30 kg/m²	3688 (29.5%)	0 (0.0%)	0 (0.0%)	1060 (92.8%)	286 (100.0%)
CrCl (recalc.), mL/min,					
mean ± SD	74.3 ± 30.42	42.6 ± 16.84	49.5 ± 17.16	108.3 ± 32.4	142.6 ± 46.92
median (Q1; Q3)	69.8 (53.0; 89.6)	39.6 (30.4; 54.2)	48.0 (37.8; 59.2)	106.5 (85.5; 128.5)	141.0 (108.4; 169.8)
<15 mL/min	3 (0.0%)	0 (0.0%)	0 (0.0%)	0 (0.0%)	0 (0.0%)
[15; 30)	288 (2.5%)	35 (24.1%)	106 (9.9%)	0 (0.0%)	0 (0.0%)
[30; 50]	2103 (18.5%)	71 (49.0%)	484 (45.2%)	19 (1.8%)	21 (1.6%)
(50; 80)	4876 (42.8%)	35 (24.1%)	429 (40.1%)	174 (17.0%)	187 (14.6%)
≥ 80 mL/min	4112 (36.1%)	4 (2.8%)	52 (4.9%)	832 (81.2%)	1069 (83.7%)
$CHADS_2$ (recalc.),					
mean ± SD	1.7 ± 1.07	179 ± 1.13	1.9 ± 1.08	1.6 ± 0.97	1.5 ± 0.87
median (Q1; Q3)	2 (1; 2)	2 (1; 2)	2 (1; 2)	1 (1; 2)	1 (1; 2)
0	1365 (10.4%)	17 (11.2%)	82 (7.1%)	125 (10.8%)	28 (9.8%)
1–2	9124 (69.7%)	107 (70.4%)	817 (70.6%)	859 (74.1%)	227 (79.1%)
≥ 3	2603 (19.9%)	28 (18.4%)	259 (22.4%)	175 (15.1%)	32 (11.1%)
CHA_2DS_2-VASc (recalc.),					
mean ± SD	3.1 ± 1.40	3.7 ± 1.36	3.9 ± 1.29	2.6 ± 1.33	2.4 ± 1.29
median (Q1; Q3)	3 (2; 4)	4 (3; 4)	4 (3; 5)	3 (2; 3)	2 (1; 3)
0	290 (2.2%)	1 (0.7%)	1 (0.1%)	42 (3.6%)	12 (4.2%)
1	1324 (10.1%)	3 (2.0%)	22 (1.9%)	200 (17.3%)	68 (23.8%)
2	2802 (21.4%)	23 (15.1%)	120 (10.4%)	313 (27.0%)	84 (29.4%)
3	3768 (28.8%)	36 (23.7%)	311 (26.9%)	331 (28.6%)	68 (23.8%)
≥ 4	4908 (37.5%)	89 (58.6%)	703 (60.8%)	272 (23.5%)	301 (18.9%)
mod. HAS-BLED (recalc.),					
mean ± SD	2.5 ± 1.10	2.5 ± 1.13	2.6 ± 1.04	2.4 ± 1.09	2.1 ± 1.05
median (Q1; Q3)	2 (2; 3)	2 (2; 3)	3 (2; 3)	2 (2; 3)	2 (1; 3)
< 3	6711 (51.3%)	75 (49.3%)	541 (46.7%)	641 (55.3%)	188 (65.5%)
≥ 3	6381 (48.7%)	77 (50.7%)	617 (53.3%)	518 (44.7%)	99 (34.5%)
Frailty *, *n* (%)					
Yes	1392 (10.6%)	59 (38.8%)	282 (24.3%)	52 (4.5%)	14 (4.9%)
No	10820 (82.7%)	85 (55.9%)	793 (68.5%)	1018 (87.9%)	252 (87.8%)

	ETNA-AF EU	Body Weight [kg]			
	Total	<50 kg	≥50 and ≤60 kg	>100 and ≤120 kg	>120 kg
Patients, N (%)	13,092 (100%)	152 (1.2%)	1158 (9.1%)	1159 (9.1%)	287 (2.3%)
Not known	878 (6.7%)	8 (5.3%)	83 (7.2%)	88 (7.6%)	21 (7.3%)
Medical history, *n* (%)					
Hypertension	10088 (77.1%)	97 (63.8%)	843 (72.8%)	963 (83.1%)	243 (84.7%)
Diabetes	2879 (22.0%)	13 (8.6%)	186 (16.1%)	378 (32.6%)	109 (38.0%)
Dys-/hyperlipidaemia	5626 (43.0%)	58 (38.2%)	429 (37.1%)	496 (42.8%)	114 (39.7%)
Coronary heart disease	2738 (20.9%)	18 (11.8%)	202 (17.4%)	260 (22.4%)	48 (16.7%)
Peripheral artery disease	437 (3.3%)	5 (3.3%)	39 (3.4%)	36 (3.1%)	10 (3.5%)
Congestive heart failure	777 (5.9%)	19 (12.5%)	170 (14.7%)	184 (15.9%)	41 (14.3%)
Myocardial infarction	560 (4.3%)	4 (2.6%)	53 (4.6%)	49 (4.2%)	8 (2.8%)
TIA	448 (3.4%)	7 (4.6%)	45 (3.9%)	28 (2.4%)	6 (2.1%)
Hyper-/hypothyroidism	1611 (12.3%)	29 (19.1%)	175 (15.1%)	130 (11.2%)	33 (11.5%)
Digestive tract disease	1086 (8.3%)	19 (12.5%)	123 (10.6%)	73 (6.3%)	22 (7.7%)
COPD	1206 (9.2%)	18 (11.8%)	107 (9.2%)	104 (9.0%)	38 (13.2%)
History of stroke & ICH, *n* (%)					
Ischaemic stroke	778 (5.9%)	13 (8.5%)	89 (7.7%)	40 (3.5%)	8 (2.8%)
Intracranial haemorrhage	62 (0.5%)	2 (1.3%)	6 (0.5%)	4 (0.4%)	0 (0.0%)
History of bleeding, *n* (%)					
Major	129 (1.0%)	3 (2.0%)	20 (1.7%)	11 (0.9%)	2 (0.7%)
Major or CRNM	270 (2.1%)	5 (3.3%)	39 (3.4%)	24 (2.1%)	5 (1.7%)
Current AF type, *n* (%)					
Paroxysmal	7039 (53.9%)	90 (59.6%)	664 (57.5%)	571 (49.4%)	142 (49.8%)
Persistent	3159 (24.2%)	32 (21.2%)	231 (20.0%)	337 (29.1%)	87 (30.5%)
Long-standing persistent & Permanent	2864 (21.9%)	29 (19.2%)	260 (22.5%)	249 (21.5%)	56 (19.6%)
Patients fulfilling ≥1 dose adjustment criteria, *n* (%)	3106 (23.7%)	152 (100.0%)	1158 (100.0%)	30 (2.6%)	5 (1.7%)
Edoxaban dose at baseline, *n* (%)	9991 (76.3%)	42 (27.6%)	446 (38.5%)	1016 (87.7%)	262 (91.3%)
60 mg	3101 (23.7%)	110 (72.4%)	712 (61.5%)	143 (12.3%)	25 (8.7%)
30 mg					

* There was no specific definition for frailty; it was left to the discretion of the physician to categorise a patient as frail. AF, atrial fibrillation; BMI, body mass index; COPD, chronic obstructive pulmonary disease; CrCl, creatinine clearance; CRNM, clinically relevant non-major; CV, cardiovascular; ICH, intracranial haemorrhage; OD, once daily; Q1, lower quartile; Q3, upper quartile; SD, standard deviation; TIA, transient ischemic attack.

Table S2. Baseline demographics and clinical characteristics of patients included in ETNA-AF-Europe categorised by BMI.

	ETNA-AF-Europe Total	Body Mass Index [kg/m²]								
		Underweight <18.5		Normal Weight ≥18.5–<25	Overweight ≥25–<30		Class 1 Obesity ≥30–<35		Class 2 + 3 Obesity ≥35	
Patients, N (%)	13092 (100)	115 (0.92)	Standardised Difference to Normal Weight	3341 (26.68)	5377 (42.94)	Standardised Difference to Normal Weight	2544 (20.32)	StandardisedDifference to Normal Weight	1144 (9.14%)	Standardised Difference to Normal Weight
Female, *n* (%)	5661 (43.2)	88 (76.5)	0.57	1680 (50.3)	2041 (38.0)	−0.25	1011 (39.7)	−0.21	602 (52.6)	0.05
Age, years										
mean ± SD	73.6 ± 9.46	76.8 ± 9.76		75.6 ± 9.38	74.0 ± 8.99		72.1 ± 9.31		68.4 ± 9.73	
median	75 (68; 80)	78 (72; 84)		77 (70; 82)	75 (69; 80)		73.0 (67; 79)		69.0 (62; 75)	
(Q1; Q3)	1994 (15.2)	10 (8.7)	0.13	362 (10.8)	724 (13.5)	−0.18	471 (18.5)	−0.37	354 (30.9)	−0.76
<65 years	4456 (34.0)	27 (23.5)		985 (29.5)	1817 (33.8)		959 (37.7)		472 (41.3)	
65–<75 years	5268 (40.2)	54 (47.0)		1466 (43.9)	2283 (42.5)		940 (36.9)		286 (25.0)	
75–<85 years	1372 (10.5)	24 (20.9)		528 (15.8)	553 (10.3)		172 (6.8)		32 (2.8)	
≥85 years										
Body weight, kg mean ± SD	81.0 ± 17.29	48.5 ± 5.58	−1.82	65.9 ± 9.34	79.5 ± 9.79	1.46	92.2 ± 11.32 92.0 (84.0; 100.0)	2.94	110.7 ± 18.16	3.12

median<br>(Q1; Q3)<br>≤60 kg<br>60–<80 kg<br>80–<100 kg<br>≥100 kg	80.0 (70.0;<br>90.0)<br>1310 (10.3)<br>5565 (43.9)<br>4346 (34.3)<br>1446 (11.4)	48.0 (45.0;<br>51.0)<br>110 (95.6)<br>5 (4.4)<br>0 (0.0)<br>0 (0.0)		65.0 (59.0;<br>72.0)<br>1066 (31.9)<br>2083 (62.4)<br>191 (5.7)<br>1 (0.0)	80.0 (72.0;<br>86.0)<br>113 (2.1)<br>2934 (54.6)<br>2249 (41.8)<br>81 (1.5)		0 (0.0)<br>459 (18.0)<br>1519 (59.7)<br>566 (22.2)		108.0 (99.0;<br>120.0)<br>0 (0.0)<br>24 (2.1)<br>340 (29.7)<br>780 (68.2)	
BMI, kg/m²<br>mean ± SD<br>median<br>(Q1; Q3)<br><18.5 kg/m²<br>18.5–<25 kg/m²<br>25–<30 kg/m²<br>30–<35 kg/m²<br>35–<40 kg/m²<br>≥40 kg/m²<br>≥30 kg/m²	28.1 ± 5.11<br>27.3 (24.7;<br>30.7)<br>115 (0.9)<br>3341 (26.7)<br>5377 (42.9)<br>2544 (20.3)<br>792 (6.3)<br>352 (2.8)<br>3688 (29.5)	17.4 ± 0.96<br>17.7 (17.0;<br>18.1)<br>115 (100.0)<br>0 (0.0)<br>0 (0.0)<br>0 (0.0)<br>0 (0.0)<br>0 (0.0)<br>0 (0.0)	−3.58	23.0 ± 1.56<br>23.4 (22.0;<br>24.2)<br>0 (0.0)<br>3341 (100.0)<br>0 (0.0)<br>0 (0.0)<br>0 (0.0)<br>0 (0.0)<br>0 (0.0)	27.3 ± 1.40<br>27.3 (26.1;<br>28.4)<br>0 (0.0)<br>0 (0.0)<br>5377 (100.0)<br>0 (0.0)<br>0 (0.0)<br>0 (0.0)<br>0 (0.0)	2.96	32.0 ± 1.42<br>31.8 (30.8; 33.2)<br>0 (0.0)<br>0 (0.0)<br>0 (0.0)<br>2544 (100.0)<br>0 (0.0)<br>0 (0.0)<br>2544 (100.0)	6.03	39.1 ± 4.27<br>37.9 (36.0; 40.7)<br>0 (0.0)<br>0 (0.0)<br>0 (0.0)<br>0 (0.0)<br>792 (69.2)<br>352 (30.8)<br>1144 (100.0)	6.35
CrCl (recalc.),<br>mL/min,<br>mean ± SD<br>median<br>(Q1; Q3)<br><15 mL/min<br>15–<30 mL/min<br>30–<50 mL/min<br>50–<80 mL/min<br>≥80 mL/min	74.3 ± 30.42<br>69.8 (53.0;<br>89.6)<br>3 (0.0)<br>288 (2.5)<br>2103 (18.5)<br>4876 (42.8)<br>4112 (36.1)	46.7 ± 21.50<br>44.1 (31.8;<br>57.1)<br>0 (0.0)<br>24 (21.1)<br>50 (43.9)<br>32 (28.1)<br>8 (7.0)	−0.61	59.8 ± 21.53<br>58.2 (44.0;<br>72.5)<br>1 (0.0)<br>163 (5.4)<br>918 (30.2)<br>1466 (48.3)<br>487 (16.0)	71.8 ± 24.44<br>69.7 (54.2;<br>86.0)<br>2 (0.0)<br>82 (1.7)<br>1545 (32.3)<br>2163 (45.2)<br>992 (20.7)	0.51	84.8 ± 29.63<br>82.3 (63.3; 102.5)<br>0 (0.0)<br>15 (0.6)<br>231 (9.1)<br>817 (32.1)<br>1213 (47.7)	0.99	108.5 ± 42.74<br>102.5 (77.6;<br>133.1)<br>0 (0.0)<br>1 (0.1)<br>58 (5.1)<br>218 (19.1)<br>760 (66.4)	1.71
CHADS₂ (recalc.),<br>mean ± SD<br>median (Q1; Q3)<br>0<br>1–2<br>≥3	1.7 ± 1.07<br>2 (1; 2)<br>1365 (10.4)<br>9124 (69.7)<br>2603 (19.9)	1.60 ± 1.10<br>2 (1; 2)<br>14 (12.2)<br>81 (70.4)<br>20 (17.4)	−0.05	1.7 ± 1.10<br>2 (1; 2)<br>421 (12.6)<br>2278 (68.2)<br>642 (19.2)	1.7 ± 1.07<br>2 (1; 2)<br>543 (10.1)<br>3771 (70.1)<br>1063 (19.8)	0.05	1.8 ± 1.04<br>2.0 (1; 2)<br>198 (7.8)<br>1794 (70.5)<br>552 (21.7)	0.09	1.7 ± 0.97<br>2.0 (1; 2)<br>90 (7.9)<br>853 (74.6)<br>201 (17.6)	−0.009
CHA₂DS₂-VASc (recalc.)<br>mean ± SD<br>median (Q1; Q3)<br>0<br>1<br>2<br>3<br>≥4	3.1 ± 1.40<br>3 (2; 4)<br>290 (2.2)<br>1324 (10.1)<br>2802 (21.4)<br>3768 (28.8)<br>4908 (37.5)	3.4 ±1.34<br>3 (3; 4)<br>1 (0.9)<br>7 (6.1)<br>18 (15.7)<br>36 (31.3)<br>53 (46.1)	0.17	3.2 ± 1.40<br>3 (2; 4)<br>82 (2.5)<br>288 (8.6)<br>686 (20.5)<br>928 (27.8)<br>1357 (40.6)	3.1 ± 1.38<br>3 (2; 4)<br>116 (2.2)<br>540 (10.0)<br>1173 (21.8)<br>1594 (29.6)<br>1954 (36.3)	−0.07	3.1 ± 1.39<br>3.0 (2.0; 4.0)<br>44 (1.7)<br>283 (11.1)<br>533 (21.0)<br>741 (29.1)<br>943 (37.1)	−0.06	3.0 ± 1.36<br>3.0 (2.0; 4.0)<br>24 (2.1)<br>138 (12.1)<br>278 (24.3)<br>311 (27.2)<br>393 (34.4)	−0.15
mod. HAS-BLED (recalc.)<br>mean ± SD<br>median (Q1; Q3)<br><3<br>≥3	2.5 ± 1.10<br>2 (2; 3)<br>6711 (51.3)<br>6381 (48.7)	2.5 ± 1.17<br>2 (2; 3)<br>63 (54.8)<br>52 (45.2)	−0.02	2.5 ± 1.11<br>2 (2; 3)<br>1738 (52.0)<br>1603 (48.0)	2.6 ± 1.10<br>3 (2; 3)<br>2639 (49.1)<br>2738 (50.9)	0.07	2.6 ± 1.09<br>3.0 (2.0; 3.0)<br>1237 (48.6)<br>1307 (51.4)	0.06	2.4 ± 1.05<br>2.0 (2.0; 3.0)<br>662 (57.9)<br>482 (42.1)	−0.12
Frailty *, *n* (%)										
Yes	1392 (10.6)	42 (36.5)	0.50	507 (15.2)	492 (9.2)	−0.19	206 (8.1)	−0.22	94 (8.2)	−0.22
No	10820 (82.7)	64 (55.7)	−0.51	2637 (78.9)	4540 (84.4)	0.14	2172 (85.4)	0.17	977 (85.4)	0.17
Not known	878 (6.7)	9 (7.8)	0.08	197 (5.9)	345 (6.4)	0.02	164 (6.4)	0.02	73 (6.4)	0.02
Medical history, *n* (%)										
Hypertension	10088 (77.1)	62 (53.9)	−0.32	2316 (69.3)	4221 (78.5)	0.21	2137 (84.0)	0.35	983 (85.9)	0.41
Diabetes	2879 (22.0)	13 (11.3)	−0.11	501 (15.0)	1095 (20.4)	0.14	752 (29.6)	0.36	412 (36.0)	0.50
Dys-/hyperlipidaemia	5626 (43.0)	42 (36.5)	−0.02	1259 (37.7)	2402 (44.7)	0.14	1205 (47.4)	0.20	502 (43.9)	0.13
Coronary heart disease	2738 (20.9)	21 (18.3)	−0.009	622 (18.2)	1224 (22.8)	0.10	578 (22.7)	0.10	210 (18.4)	−0.007

Peripheral artery disease	437 (3.3)	6 (5.2)	0.09	115 (3.4)	189 (3.5)	0.004	83 (3.3)	−0.01	28 (2.4)	−0.06
Congestive heart failure	1850 (14.2)	19 (16.5)	0.04	455 (13.6)	757 (14.1)	0.009	380 (15.0)	0.03	181 (15.9)	0.10
Myocardial infarction	560 (4.3)	4 (3.5)	−0.04	144 (4.3)	244 (4.5)	0.01	120 (4.7)	0.02	26 (2.3)	−0.11
TIA	448 (3.4)	5 (4.3)	0.02	135 (4.0)	167 (3.1)	−0.05	78 (3.1)	−0.05	26 (2.3)	−0.10
Hyper /hypothyroidism	1611 (12.3)	13 (11.3)	−0.02	398 (11.9)	634 (11.8)	−0.004	314 (12.3)	0.01	187 (16.3)	0.13
Digestive tract disease	1086 (8.3)	19 (16.5)	0.24	287 (8.6)	439 (8.2)	−0.02	215 (8.5)	−0.005	84 (7.3)	−0.05
COPD	1206 (9.2)	15 (13.0)	0.14	287 (8.6)	491 (9.1)	0.02	256 (10.1)	0.05	126 (11.0)	0.08
History of stroke & ICH, *n* (%)										
Ischaemic stroke	778 (5.9)	9 (7.8)	0.05	222 (6.6)	324 (6.0)	−0.03	130 (5.1)	−0.07	35 (3.1)	−0.17
Intracranial haemorrhage	62 (0.5)	1 (0.9)	0.06	13 (0.4)	35 (0.7)	0.04	10 (0.4)	0.0006	1 (0.1)	−0.06
History of bleeding, *n* (%)	424 (3.2)	4 (3.5)	−0.02	129 (3.9)	171 (3.2)	−0.04	66 (2.6)	−0.07	38 (3.3)	−0.03
Major	129 (1.0)	1 (0.9)	−0.04	41 (1.2)	52 (1.0)	−0.03	19 (0.7)	−0.05	12 (1.0)	−0.02
Major or CRNM	270 (2.1)	3 (2.6)	0.006	84 (2.5)	111 (2.1)	−0.03	42 (1.7)	−0.06	21 (1.8)	−0.05
Current AF type, *n* (%)										
Paroxysmal	7039 (53.9)	66 (57.9)	0.002	1915 (57.4)	2845 (53.0)	−0.09	1303 (51.2)	−0.12	566 (49.5)	−0.16
Persistent	3159 (24.2)	27 (23.7)	0.04	729 (21.9)	1345 (25.0)	0.08	657 (25.8)	0.09	305 (26.7)	0.11
Long-standing persistent & Permanent	2864 (21.9)	21 (18.4)	−0.06	692 (20.7)	1180 (22.0)	0.03	583 (22.9)	0.05	271 (23.7)	0.07
Patients fulfilling ≥1 dose adjustment criteria, *n* (%)	3106 (23.7)	112 (97.4)	1.32	1622 (48.5)	977 (18.2)	−0.68	269 (10.6)	−0.92	73 (6.4)	−1.07
Edoxaban dose at baseline, *n* (%)<br>60 mg OD<br>30 mg OD	9991 (76.3)<br>3101 (23.7)	43 (37.4)<br>72 (62.6)	−0.57<br>0.57	2170 (65.0)<br>1171 (35.0)	4279 (79.6)<br>1098 (20.4)	0.33<br>−0.33	2081 (81.8)<br>463 (18.2)	0.39<br>−0.39	968 (84.6)<br>176 (15.4)	0.46<br>−0.46

* There was no specific definition for frailty; it was left to the discretion of the physician to categorise a patient as frail. AF, atrial fibrillation; BMI, body mass index; COPD, chronic obstructive pulmonary disease; CrCl, creatinine clearance; CRNM, clinically relevant non-major; CV, cardiovascular; ICH, intracranial haemorrhage; OD, once daily; Q1, lower quartile; Q3, upper quartile; SD, standard deviation; TIA, transient ischemic attack.

Table S3. Outcomes at 1 year split by gender for weight categories (unadjusted data).

Event Type	Gender	Weight (kg)–HR Compared with Normal Weight >60 and ≤80kg			
		≤60 kg	>60–≤80 kg	>80–≤100 kg	>100 kg
Major bleeding	Female	1.15 (0.64,2.07) *p* = 0.65	1.00-reference	0.51 (0.23,1.15) *p* = 0.10	0.75 (0.23,2.45) *p* = 0.64
	Male	0.00 (0.00, NE) *p* = 0.98	1.00-reference	0.78 (0.48,1.29) *p* = 0.33	0.22 (0.07,0.71) *p* = 0.0116
Major or CRNM bleeding	Female	1.01 (0.65,1.56) *p* = 0.98	1.00-reference	0.77 (0.47,1.25) *p* = 0.29	0.63 (0.25,1.55) *p* = 0.31
	Male	0.71 (0.22,2.26) *p* = 0.56	1.00-reference	0.77 (0.55,1.08) *p* = 0.13	0.55 (0.33,0.94) *p* = 0.0287
Major GI bleeding	Female	1.65 (0.64,4.24) *p* = 0.30	1.00-reference	0.96 (0.31,3.02) *p* = 0.94	0.00 (0.00, NE) *p* = 0.99
	Male	0.00 (0.00, NE) *p* = 0.99	1.00-reference	0.96 (0.31,3.02) *p* = 0.94	0.76 (0.15,3.89) *p* = 0.74
Intracranial haemorrhage	Female	1.29 (0.24,7.06) *p* = 0.77	1.00-reference	1.98 (0.44,8.84) *p* = 0.37	2.27 (0.25,20.28) *p* = 0.46
	Male	0.00 (0.00, NE) *p* = 0.99	1.00-reference	0.72 (0.28,1.86) *p* = 0.50	0.26 (0.03,2.04) *p* = 0.20
Any stroke or systemic embolism	Female	0.92 (0.46,1.82) *p* = 0.81	1.00-reference	0.94 (0.47,1.86) *p* = 0.85	0.29 (0.04,2.13) *p* = 0.22
	Male	0.00 (0.00, NE) *p* = 0.98	1.00-reference	1.00 (0.53,1.90) *p* = 1.00	0.96 (0.40,2.31) *p* = 0.92
Ischaemic stroke	Female	0.90 (0.40,2.01) *p* = 0.79	1.00-reference	0.57 (0.22,1.50) *p* = 0.26	0.39 (0.05,2.90) *p* = 0.36
	Male	0.00 (0.00, NE) *p* = 0.99	1.00-reference	0.81 (0.36,1.80) *p* = 0.61	0.97 (0.34,2.75) *p* = 0.95
Haemorrhagic stroke	Female	2.61 (0.16,41.66) *p* = 0.50	1.00-reference	5.28 (0.48,58.3) *p* = 0.17	0.00 (0.00, NE) *p* = 1.00
	Male	0.00 (0.00, NE) *p* = 0.99	1.00-reference	1.02 (0.27,3.78) *p* = 0.98	0.59 (0.07,5.24) *p* = 0.63
	Female	2.60 (0.91,7.42) *p* = 0.07	1.00-reference	3.40 (1.27,9.13) *p* = 0.0152	1.29 (0.16,10.48) *p* = 0.81

Myocardial infarction	Male	1.04 (0.14,7.84) *p* = 0.97	1.00-reference	1.07 (0.56,2.04) *p* = 0.85	0.29 (0.07,1.27) *p* = 0.10
Cardiovascular mortality (sensitivity analysis)	Female	1.48 (0.90,2.44) *p* = 0.13	1.00-reference	0.82 (0.44,1.52) *p* = 0.52	0.86 (0.31,2.40), *p* = 0.77
	Male	3.06 (1.56,6.01) *p* = 0.0011	1.00-reference	0.59 (0.39,0.89) *p* = 0.0108	0.55 (0.30,1.01) *p* = 0.05
All-cause mortality	Female	2.19 (1.58,3.04) *p* < 0.0001	1.00-reference	1.08 (0.72,1.63) *p* = 0.71	0.81 (0.37,1.76) *p* = 0.59
	Male	2.93 (1.80,4.77) *p* < 0.0001	1.00-reference	0.64 (0.48,0.85) *p* = 0.0018	0.63 (0.42,0.95) *p* = 0.0273

CRNM, clinically relevant non-major bleeding; GI, gastrointestinal; ICH, intracranial haemorrhage; NE, non-estimable.

Table S4. Outcomes at 1 year split by gender for weight categories (adjusted for eGFR and CHA_2DS_2-VASc).

Event Type	Gender N (Total Events; Events Per Group)	Weight (kg)–HR Compared with Normal Weight >60 and ≤80kg			
		≤60 kg	>60–≤80 kg	>80–≤100 kg	>100 kg
Major bleeding	Female (62; 16, 36, 7, 3)	0.99 (0.54,1.84) *p* = 0.98	1.00-reference	0.62 (0.27,1.43) *p* = 0.26	1.32 (0.36,4.80) *p* = 0.68
	Male (70; 0, 32, 31, 3)	0.00 (0.00, NE) *p* = 0.98	1.00-reference	1.02 (0.60,1.74) *p* = 0.94	0.43 (0.12,1.53) *p* = 0.19
Major or CRNM bleeding	Female (129; 28, 72, 21, 5)	0.92 (0.58,1.45) *p* = 0.71	1.00-reference	0.95 (0.57,1.59) *p* = 0.84	0.96 (0.33,2.81) *p* = 0.94
	Male (164; 3, 71, 68, 17)	0.55 (0.17,1.77) *p* = 0.32	1.00-reference	0.98 (0.68,1.40) *p* = 0.91	0.87 (0.45,1.67) *p* = 0.67
Major GI bleeding	Female (22; 7, 11, 4, 0)	1.28 (0.47,3.47) *p* = 0.63	1.00-reference	1.22 (0.37,4.01) *p* = 0.74	0.00 (0.00, NE) *p* = 0.99
	Male (29; 0, 14, 11, 2)	0.00 (0.00, NE) *p* = 0.99	1.00-reference	0.89 (0.39,2.06) *p* = 0.79	0.76 (0.15,3.89) *p* = 0.74
Intracranial haemorrhage	Female (10; 2, 4, 3, 1)	1.60 (0.25,10.01) *p* = 0.62	1.00-reference	2.77 (0.53,14.58) *p* = 0.23	3.58 (0.26,50.01) *p* = 0.34
	Male (20; 0, 9, 8, 1)	0.00 (0.00, NE) *p* = 0.99	1.00-reference	0.89 (0.31,2.55) *p* = 0.83	0.36 (0.04,3.64) *p* = 0.39
Any stroke or systemic embolism	Female (55; 11, 31, 11, 1)	0.81 (0.37,1.76) *p* = 0.59	1.00-reference	1.04 (0.49,2.22) p = 0.92	0.44 (0.05,3.60) *p* = 0.44
	Male (48; 0, 17, 21, 7)	0.00 (0.00, NE) *p* = 0.98	1.00-reference	1.24 (0.62,2.47) *p* = 0.54	1.63 (0.58,4.64) *p* = 0.36
Ischaemic stroke	Female (38; 8, 23, 5, 1)	0.76 (0.30,1.93) *p* = 0.56	1.00-reference	0.60 (0.22,1.64) *p* = 0.32	0.40 (0.04,3.59) *p* = 0.41
	Male (32; 0, 12, 12, 5)	0.00 (0.00, NE) *p* = 0.99	1.00-reference	0.91 (0.38,2.18) *p* = 0.83	1.36 (0.38,4.87) *p* = 0.64
Haemorrhagic stroke	Female (4; 1, 1, 2, 0)	0.00 (0.00, NE) *p* = 01.00	1.00-reference	2.42 (0.34,17.2) *p* = 0.36	0.00 (0.00, NE) *p* = 1.00
	Male (10; 0, 4, 5, 1)	0.00 (0.00, NE) *p* = 0.99	1.00-reference	1.13 (0.28,4.55) *p* = 0.86	0.73 (0.06,9.08) *p* = 0.81
Myocardial infarction	Female (25; 7, 7, 9, 1)	2.57 (0.87,7.61) *p* = 0.09	1.00-reference	3.40 (1.21,9.58) *p* = 0.0208	2.16 (0.27,17.28) *p* = 0.60
	Male (41; 1, 16, 21, 2)	0.92 (0.12,7.06) *p* = 0.93	1.00-reference	1.38 (0.67,2.87) *p* = 0.39	0.57 (0.11,2.86) *p* = 0.50
Cardiovascular mortality (sensitivity analysis)	Female (85; 24, 42, 13, 4)	1.25 (0.73,2.15) *p* = 0.42	1.00-reference	1.41 (0.73,2.70) p = 0.31	2.42 (0.69,8.43) *p* = 0.17
	Male 1(21; 10, 55, 40, 13)	2.18 (1.09,4.35) *p* = 0.0268	1.00-reference	0.84 (0.54,1.30) *p* = 0.43	1.00 (0.47,2.13) *p* = 1.00
All-cause mortality	Female (183; 66, 78, 32, 7 *)	1.87 (1.31,2.67) *p* = 0.0006	1.00-reference	1.66 (1.07,2.57) *p* = 0.0244	2.16 (0.89,5.24) p = 0.09
	Male (246; 19, 110, 87, 30 *)	2.00 (1.22,3.28) *p* = 0.0063	1.00-reference	0.91 (0.68,1.24) *p* = 0.56	1.49 (0.91,2.42) *p* = 0.11

* 4 deaths at females and 8 deaths at males with missing weight. CRNM, clinically relevant non-major bleeding; GI, gastrointestinal; ICH, intracranial haemorrhage; NE, non-estimable.

Table S5. Outcomes at 1-year categorised by BMI (unadjusted data).

Annualised Event Rates, *n* (%/Year) [95% CI]	Etna-af-Europe Total	BMI [kg/m²]					BMI [kg/m²]–HR Compared with Normal Weight BMI ≥18.5 and <25 Unadjusted HRs				
		Underweight <18.5 kg/m²	Normal Weight ≥18.5–<25 kg/m²	Overweight ≥25–<30 kg/m²	Class 1 Obesity ≥30–<30 kg/m²	Class 2+3 Obesity ≥35 kg/m²	Underweight <18.5 kg/m²	Normal Weight ≥18.5<25 kg/m²	Overweight ≥25 –d <30 kg/m²	Class 1 Obesity ≥30–<35 kg/m²	Class 2+3 Obesity ≥35 kg/m²
Patients, N (%)	13092 (100%)	115 (0.92%)	3341 (26.68%)	5377 (42.94%)	2544 (20.32%)	1144 (9.14%)	115 (0.92%)	3341 (26.68%)	5377 (42.94%)	2544 (20.32%)	1144 (9.14%)
Major bleeding	132 (1.05) [0.88; 1.25]	0 (0.00) n.a.	41 (1.29) [0.92; 1.75]	57 (1.10) [0.83; 1.43]	22 (0.89) [0.53;1.25]	7 (0.64) [0.19; 1.09]	0.00 (0.00, NE) *p* = 0.97	1.00-reference	0.86 (0.57, 1.28) *p* = 0.45	0.70 (0.41, 1.17) *p* = 0.17	0.50 (0.22, 1.10) *p* = 0.09
Major or CRNM bleeding	293 (2.35) [2.09; 2.63]	5 (4.91) [1.59; 11.44]	88 (2.79) [2.23; 3.43]	110 (2.14) [1.76; 2.58]	58 (2.37) [1.79; 2.95]	21 (1.92) [1.14; 2.69]	1.76 (0.71, 4.32) *p* = 0.22	1.00-reference	0.77 (0.58, 1.02) *p* = 0.07	0.85 (0.61, 1.19) *p* = 0.34	0.69 (0.43, 1.11) *p* = 0.13
Major GI bleeding	51 (0.40) [0.30; 0.53]	0 (0.00) n.a.	15 (0.47) [0.26; 0.77]	21 (0.40) [0.25; 0.62]	9 (0.36) [0.13; 0.59]	3 (0.27) [0.00; 0.57]	0.00 (0.00, NE) *p* = 0.98	1.00-reference	0.86 (0.45, 1.68) *p* = 0.67	0.78 (0.34, 1.78) *p* = 0.55	0.58 (0.17, 2.01) *p* = 0.39
Intracranial haemorrhage	30 (0.24) [0.16; 0.34]	0 (0.00) n.a.	7 (0.22) [0.09; 0.45]	14 (0.27) [0.15; 0.45]	5 (0.20) [0.02; 0.37]	2 (0.18) [0.00; 0.43]	0.00 (0.00, NE) *p* = 0.99	1.00-reference	1.23 (0.50, 3.05) *p* = 0.65	0.93 (0.29, 2.92) *p* = 0.90	0.83 (0.17, 3.99) *p* = 0.82
Any stroke or systemic embolism	103 (0.82) [0.67; 0.99]	1 (0.97) [0.02; 5.41]	22 (0.69) [0.43; 1.04]	47 (0.91) [0.67; 1.21]	17 (0.69) [0.37; 1.00]	12 (1.09) [0.50; 1.68]	1.40(0.19,10.39) *p* = 0.74	1.00-reference	1.32 (0.79, 2.19) *p* = 0.29	1.00 (0.53, 1.89) *p* = 0.99	1.58 (0.78, 3.20) *p* = 0.20

Ischaemic stroke	70 (0.56) [0.43; 0.70]	1 (0.97) [0.02; 5.41]	17 (0.53) [0.31; 0.85]	32 (0.62) [0.42; 0.87]	8 (0.32) [0.11; 0.54]	8 (0.73) [0.25; 1.21]	1.81(0.24,13.60) *p* = 0.56	1.00-reference	1.16 (0.65, 2.09) *p* = 0.62	0.61 (0.26, 1.41) *p* = 0.25	1.36 (0.59, 3.16) *p* = 0.47
Haemorrhagic stroke	14 (0.11) [0.06; 0.19]	0 (0.00) n.a.	3 (0.09) [0.02; 0.27]	6 (0.12) [0.04; 0.25]	4 (0.16) [0.00; 0.31]	1 (0.09) [0.00; 0.26]	0.00 (0.00, NE) *p* = 0.99	1.00-reference	1.23 (0.31, 4.93) *p* = 0.77	1.73 (0.39,7.75) *p* = 0.47	0.97(0.10,9.30) *p* = 0.98
Myocardial infarction	66 (0.52) [0.41; 0.67]	0 (0.00) n.a.	13 (0.41) [0.22; 0.70]	32 (0.62) [0.42; 0.87]	12 (0.49) [0.22; 0.70]	6 (0.54) [0.23; 0.76]	0.00 (0.00, NE) *p* = 0.98	1.00-reference	1.52 (0.80, 2.90) *p* = 0.20	1.20 (0.55, 2.63) *p* = 0.65	1.34 (0.51, 3.52) *p* = 0.55
Cardiovascular mortality (sensitivity analysis)	206 (1.63) [1.42; 1.87]	7 (6.75) [2.71; 13.87]	63 (1.97) [1.51; 2.52]	80 (1.54) [1.22; 1.92]	33 (1.34) [0.90; 1.78]	15 (1.36) [0.70; 2.02]	3.43 (1.57, 7.49) *p* = 0.0020	1.00-reference	0.78 (0.56, 1.10) *p* = 0.15	0.68 (0.45, 1.04) *p* = 0.07	0.69 (0.39, 1.21) *p* = 0.20
All-cause mortality	442 (3.50) [3.18; 3.84]	16 (15.43) [8.79; 24.98]	143 (4.46) [3.76; 5.26]	160 (3.08) [2.62; 3.59]	64 (2.59) [1.98; 3.19]	36 (3.26) [2.25; 4.27]	3.47 (2.07, 5.81) p<0.0001	1.00-reference	0.69 (0.55, 0.86) *p* = 0.0012	0.58 (0.43, 0.78) *p* = 0.0003	0.73 (0.51, 1.05) *p* = 0.09

Table S6. Outcomes at 1-year categorised by BMI (adjusted for eGFR and CHA_2DS_2-VASc).

Annualised Event Rates, *n* (%/Year) [95% CI]	ETNA-AF-Europe Total	BMI [kg/m²]					BMI [kg/m²]–HR Compared with Normal Weight BMI ≥18.5 and <25				
		Underweight <18.5 kg/m²	Normal Weight ≥18.5–<25 kg/m²	Overweight ≥25–<30 kg/m²	Class 1 Obesity ≥30–<30 kg/m²	Class 2+3 Obesity ≥35 kg/m²	Underweight <18.5 kg/m²	Normal Weight ≥18.5–<25 kg/m²	Overweight ≥25–<30 kg/m²	Class 1 Obesity ≥30–<35 kg/m²	Class 2+3 Obesity ≥35 kg/m²
Patients, N (%)	13092 (100%)	115 (0.92%)	3341 (26.68%)	5377 (42.94%)	2544 (20.32%)	1144 (9.14%)	115 (0.92%)	3341 (26.68%)	5377 (42.94%)	2544 (20.32%)	1144 (9.14%)
Major bleeding	132 (1.05) [0.88; 1.25]	0 (0.00) n.a.	41 (1.29) [0.92; 1.75]	57 (1.10) [0.83; 1.43]	22 (0.89) [0.53; 1.25]	7 (0.64) [0.19; 1.09]	0.00 (0.00, NE) *p* = 0.97	1.00-reference	0.99 (0.65, 1.50) *p* = 0.96	0.93 (0.54, 1.60) *p* = 0.78	0.83 (0.35, 1.95) *p* = 0.66
Major or CRNM bleeding	293 (2.35) [2.09; 2.63]	5 (4.91) [1.59; 11.44]	88 (2.79) [2.23; 3.43]	110 (2.14) [1.76; 2.58]	58 (2.37) [1.79; 2.95]	21 (1.92) [1.14; 2.69]	1.40 (0.57, 3.46) *p* = 0.47	1.00-reference	0.88 (0.65, 1.18) *p* = 0.38	1.01 (0.70, 1.46) *p* = 0.95	1.01 (0.59, 1.74) *p* = 0.97
Major GI bleeding	51 (0.40) [0.30; 0.53]	0 (0.00) n.a.	15 (0.47) [0.26; 0.77]	21 (0.40) [0.25; 0.62]	9 (0.36) [0.13; 0.59]	3 (0.27) [0.00; 0.57]	0.00 (0.00, NE) *p* = 0.98	1.00-reference	1.08 (0.55, 2.13) *p* = 0.82	1.17 (0.49, 2.79) *p* = 0.73	1.20 (0.32, 4.48) *p* = 0.79
Intracranial haemorrhage	30 (0.24) [0.16; 0.34]	0 (0.00) n.a.	7 (0.22) [0.09; 0.45]	14 (0.27) [0.15; 0.45]	5 (0.20) [0.02; 0.37]	2 (0.18) [0.00; 0.43]	0.00 (0.00, NE) *p* = 0.99	1.00-reference	1.38 (0.52, 3.70) *p* = 0.52	1.10 (0.32, 3.84) *p* = 0.88	1.00 (0.17, 5.86) *p* = 1.00
Any stroke or systemic embolism	103 (0.82) [0.67; 0.99]	1 (0.97) [0.02; 5.41]	22 (0.69) [0.43; 1.04]	47 (0.91) [0.67; 1.21]	17 (0.69) [0.37; 1.00]	12 (1.09) [0.50; 1.68]	1.18 (0.16, 8.82) *p* = 0.87	1.00-reference	1.43 (0.83, 2.45) *p* = 0.20	1.20 (0.60, 2.37) *p* = 0.61	2.12 (0.94, 4.80) *p* = 0.07
Ischaemic stroke	70 (0.56) [0.43; 0.70]	1 (0.97) [0.02; 5.41]	17 (0.53) [0.31; 0.85]	32 (0.62) [0.42; 0.87]	8 (0.32) [0.11; 0.54]	8 (0.73) [0.25; 1.21]	1.65(0.22,12.55) *p* = 0.63	1.00-reference	1.23 (0.65, 2.32) *p* = 0.52	0.60 (0.24, 1.53) *p* = 0.28	1.59 (0.60, 4.22) *p* = 0.35
Haemorrhagic stroke	14 (0.11) [0.06; 0.19]	0 (0.00) n.a.	3 (0.09) [0.02; 0.27]	6 (0.12) [0.04; 0.25]	4 (0.16) [0.00; 0.31]	1 (0.09) [0.00; 0.26]	0.00 (0.00, NE) *p* = 0.99	1.00-reference	1.13 (0.26, 4.85) *p* = 0.87	2.05 (0.42,10.14) *p* = 0.38	1.29(0.11,15.21) *p* = 0.84
Myocardial infarction	66 (0.52) [0.41; 0.67]	0 (0.00) n.a.	13 (0.41) [0.22; 0.70]	32 (0.62) [0.42; 0.87]	12 (0.49) [0.22; 0.70]	6 (0.54) [0.23; 0.76]	0.00 (0.00, NE) *p* = 0.98	1.00-reference	1.71 (0.87, 3.38) *p* = 0.12	1.39 (0.59, 3.27) *p* = 0.45	1.64 (0.53, 5.11) *p* = 0.39
Cardiovascular mortality (sensitivity analysis)	206 (1.63) [1.42; 1.87]	7 (6.75) [2.71; 13.87]	63 (1.97) [1.51; 2.52]	80 (1.54) [1.22; 1.92]	33 (1.34) [0.90; 1.78]	15 (1.36) [0.70; 2.02]	2.56 (1.16, 5.64) *p* = 0.0194	1.00-reference	1.01 (0.71, 1.44) *p* = 0.95	0.98 (0.61, 1.55) *p* = 0.91	1.29 (0.68, 2.46) *p* = 0.44
All-cause mortality	442 (3.50) [3.18; 3.84]	16 (15.43) [8.79; 24.98]	143 (4.46) [3.76; 5.26]	160 (3.08) [2.62; 3.59]	64 (2.59) [1.98; 3.19]	36 (3.26) [2.25; 4.27]	2.51 (1.49,4.22) *p* = 0.0006	1.00-reference	0.87 (0.69, 1.10) *p* = 0.24	0.79 (0.57, 1.10) *p* = 0.16	1.44 (0.95, 2.17) *p* = 0.08

Table S7. Outcomes at 1-year split by gender for BMI categories (unadjusted data).

Event Type	Gender	BMI [kg/m²]–HR Compared with Normal Weight BMI ≥18.5 and <25				
		Underweight <18.5 kg/m²	Normal Weight ≥18.5–<25 kg/m²	Overweight ≥25–<30 kg/m²	Class 1 Obesity ≥30–<35kg/m²	Class 2+3 Obesity ≥35 kg/m²
Major bleeding	Female	0.00 (0.00, NE) *p* = 0.98	1.00-reference	0.67 (0.38, 1.17) *p* = 0.16	0.55 (0.26,1.16) *p* = 0.12	0.41 (0.14,1.17) *p* = 0.10
	Male	0.00 (0.00, NE) *p* = 0.98	1.00-reference	1.23 (0.66, 2.29) *p* = 0.51	0.99 (0.47,2.11) *p* = 0.99	0.65 (0.19,2.27) *p* = 0.50
Major or CRNM bleeding	Female	1.61 (0.58,4.45) *p* = 0.36	1.00-reference	0.62 (0.40, 0.95) *p* = 0.0265	0.70 (0.42,1.17) *p* = 0.18	0.79 (0.44,1.44) *p* = 0.45
	Male	1.86 (0.26,13.56) *p* = 0.54	1.00-reference	0.92 (0.62, 1.36) *p* = 0.68	1.01 (0.65,1.59) *p* = 0.96	0.54 (0.24,1.21) *p* = 0.14
Major GI bleeding	Female	0.00 (0.00, NE) *p* = 0.99	1.00-reference	0.45 (0.16, 1.21) *p* = 0.11	0.60 (0.19,1.88) *p* = 0.38	0.25 (0.03,1.95) *p* = 0.19
	Male	0.00 (0.00, NE) *p* = 0.99	1.00-reference	1.86 (0.61, 5.59) *p* = 0.27	1.34 (0.36,4.99) *p* = 0.66	1.52 (0.28,8.30) *p* = 0.63
Intracranial haemorrhage	Female	0.00 (0.00, NE) *p* = 0.99	1.00-reference	1.09 (0.24, 4.88) *p* = 0.91	1.10 (0.18,6.57) *p* = 0.92	0.93 (0.10,8.90) *p* = 0.95
	Male	0.00 (0.00, NE) *p* = 0.99	1.00-reference	1.23 (0.39, 3.93) *p* = 0.72	0.80 (0.18,3.58) *p* = 0.77	0.76 (0.09,6.79) *p* = 0.80
Any stroke or systemic embolism	Female	1.31 (0.17,9.93) *p* = 0.79	1.00-reference	1.37 (0.72, 2.59) *p* = 0.34	0.99 (0.43,2.26) *p* = 0.98	0.74 (0.25,2.22) *p* = 0.59
	Male	0.00 (0.00, NE) *p* = 0.99	1.00-reference	1.55 (0.66, 3.63) *p* = 0.31	1.22 (0.44,3.37) *p* = 0.70	3.48 (1.26,9.60) *p* = 0.0160
Ischaemic stroke	Female	1.64 (0.21,12.60) *p* = 0.64	1.00-reference	1.23 (0.59, 2.56) *p* = 0.58	0.41 (0.12,1.45) *p* = 0.17	0.69 (0.20,2.45) *p* = 0.57
	Male	0.00 (0.00, NE) *p* = 0.99	1.00-reference	1.38 (0.50, 3.84) *p* = 0.53	1.07 (0.31,3.70) *p* = 0.92	3.04 (0.88,10.50) *p* = 0.08
Haemorrhagic stroke	Female	0.00 (0.00, NE) *p* = 1.00	1.00-reference	1.63 (0.15, 17.99) *p* = 0.69	1.64 (0.10,26.22) *p* = 0.73	0.00 (0.00, NE) *p* = 1.00
	Male	0.00 (0.00, NE) *p* = 0.99	1.00-reference	0.99 (0.18, 5.40) *p* = 0.99	1.61 (0.27,9.65) *p* = 0.60	1.52 (0.14,16.81) *p* = 0.73
Myocardial infarction	Female	0.00 (0.00, NE) *p* = 0.99	1.00-reference	1.10 (0.38, 3.14) *p* = 0.87	1.37 (0.42,4.48) *p* = 0.61	2.32 (0.71,7.60) *p* = 0.16
	Male	0.00 (0.00, NE) *p* = 0.99	1.00-reference	1.70 (0.73, 3.94) *p* = 0.22	1.07 (0.38,3.06) *p* = 0.89	0.43 (0.05,3.53) *p* = 0.44
Cardiovascular mortality (sensitivity analysis)	Female	1.19 (0.29,4.96) *p* = 0.81	1.00-reference	0.65 (0.39, 1.08) *p* = 0.09	0.70 (0.37,1.30) *p* = 0.26	0.50 (0.21,1.20) *p* = 0.12
	Male	12.11 (4.70,31.22) *p* < 0.0001	1.00-reference	0.89 (0.57, 1.39) *p* = 0.60	0.68 (0.38,1.20) *p* = 0.18	0.91 (0.43,1.92) *p* = 0.80
All-cause mortality	Female	1.81 (0.84,3.93) *p* = 0.13	1.00-reference	0.58 (0.41, 0.82) *p* = 0.0023	0.52 (0.33,0.82) *p* = 0.0050	0.66 (0.39,1.10) *p* = 0.11
	Male	9.92 (4.95,19.90) *p* < 0.0001	1.00-reference	0.78 (0.57, 1.06) *p* = 0.11	0.62 (0.42,0.92) *p* = 0.0184	0.81 (0.48,1.37) *p* = 0.44

Table S8. Outcomes at 1-year split by gender for BMI categories (adjusted for eGFR and CHA_2DS_2-VASc).

Event Type	Gender N (Total Events; Events Per Group)	BMI [kg/m²]–HR Compared with Normal Weight BMI ≥18.5 and <25				
		Underweight <18.5 kg/m²	Normal Weight ≥18.5–<25 kg/m²	Overweigh ≥25–<30 kg/m²	Class 1 Obesity ≥30–<35 kg/m²	Class 2+3 Obesity ≥35 kg/m²
Major bleeding	Female (62; 0, 27, 22, 9, 4)	0.00 (0.00, NE) *p* = 0.98	1.00-reference	0.69 (0.38, 1.23) *p* = 0.20	0.64 (0.29,1.41) *p* = 0.27	0.59 (0.19,1.83) *p* = 0.36
	Male (70; 0, 14, 35, 13, 3)	0.00 (0.00, NE) *p* = 0.98	1.00-reference	1.52 (0.80, 2.92) *p* = 0.20	1.45 (0.65,3.26) *p* = 0.37	1.39 (0.37,5.23) *p* = 0.63
Major or CRNM bleeding	Female (129; 4, 49, 37, 21, 14)	1.39 (0.50,3.88) *p* = 0.53	1.00-reference	0.66 (0.42, 1.03) *p* = 0.07	0.75 (0.42,1.32) *p* = 0.31	1.16 (0.58,2.29) *p* = 0.68
	Male (164; 1, 39, 73, 37, 7)	1.37 (0.19,10.04) *p* = 0.75	1.00-reference	1.05 (0.70, 1.58) *p* = 0.8100	1.26 (0.77,2.07) *p* = 0.36	0.84 (0.34,2.10) *p* = 0.71
Major GI bleeding	Female (22; 0, 11, 6, 4, 1)	0.00 (0.00, NE) *p* = 0.99	1.00-reference	0.52 (0.19, 1.44) *p* = 0.21	0.81 (0.24,2.74) *p* = 0.74	0.47 (0.05,4.07) *p* = 0.49
	Male (2; 0, 4, 15, 5, 2)	0.00 (0.00, NE) *p* = 0.99	1.00-reference	2.34 (0.77, 7.15) *p* = 0.14	2.10 (0.54,8.22) *p* = 0.29	3.83 (0.63,23.23) *p* = 0.14
Intracranial haemorrhage	Female (10; 0, 3, 4, 2, 1)	0.00 (0.00, NE) *p* = 0.99	1.00-reference	0.79 (0.16, 4.03) *p* = 0.78	1.01 (0.15,6.69) *p* = 0.99	0.78 (0.06,10.45) *p* = 0.85
	Male (20; 0, 4, 10, 3, 1)	0.00 (0.00, NE) *p* = 0.99	1.00-reference	1.75 (0.47, 6.48) *p* = 0.40	1.16 (0.22,6.21) *p* = 0.86	1.35 (0.12,15.68) *p* = 0.81
Any stroke or systemic embolism	Female (55; 1, 15, 25, 9, 4)	1.39 (0.18,10.65) *p* = 0.75	1.00-reference	1.29 (0.64, 2.60) *p* = 0.48	1.12 (0.46,2.73) *p* = 0.80	0.72 (0.18,2.80) p = 0.63
	Male (48; 0, 7, 22, 8, 8)	0.00 (0.00, NE) *p* = 0.99	1.00-reference	1.69 (0.71, 4.01) *p* = 0.24	1.37 (0.46,4.05) *p* = 0.57	5.91 (1.90,18.36) *p* = 0.0021
Ischaemic stroke	Female (38; 1, 12, 18, 3, 3)	1.93 (0.25,15.15) *p* = 0.53	1.00-reference	1.20 (0.54, 2.67) *p* = 0.66	0.42 (0.11, 1.60) *p* = 0.21	0.71 (0.16,3.08) *p* = 0.64
	Male (32; 0, 5, 14, 5, 5)	0.00 (0.00, NE) *p* = 0.99	1.00-reference	1.36 (0.48, 3.88) *p* = 0.56	0.93 (0.24,3.65) *p* = 0.92	4.00 (0.97,16.51) *p* = 0.06
Haemorrhagic stroke	Female (4; 0, 1, 2, 1, 0)	0.00 (0.00, NE) *p* = 1.00	1.00-reference	1.61 (0.15, 17.75) *p* = 0.70	1.57 (0.10,25.34) *p* = 0.75	0.00 (0.00, NE) *p* = 1.00

	Male (10; 0, 2, 4, 3, 1)	0.00 (0.00, NE) *p* = 0.99	1.00-reference	1.05 (0.19, 5.75) *p* = 0.96	1.84 (0.30,11.23) *p* = 0.51	2.00 (0.17,23.47) *p* = 0.58
Myocardial infarction	Female (25; 0, 6, 8, 5, 5)	0.00 (0.00, NE) *p* = 0.99	1.00-reference	1.10 (0.38, 3.21) *p* = 0.86	1.45 (0.42,4.98) *p* = 0.55	2.47 (0.59,10.27) *p* = 0.21
	Male (41; 0, 7, 24, 7, 1)	0.00 (0.00, NE) *p* = 0.99	1.00-reference	2.02 (0.81, 5.05) *p* = 0.13	1.30 (0.40,4.26) *p* = 0.66	0.77 (0.08,7.07) *p* = 0.82
Cardiovascular mortality (sensitivity analysis)	Female (85; 2, 33, 26, 14, 6)	1.02 (0.24,4.30) *p* = 0.98	1.00-reference	0.75 (0.43, 1.32) *p* = 0.32	1.10 (0.56,2.14) *p* = 0.78	1.05 (0.38,2.88) *p* = 0.92
	Male (121; 5, 30, 54, 19, 9)	7.67 (2.92,20.12) *p* < 0.0001	1.00-reference	1.13 (0.71, 1.80) *p* = 0.62	0.88 (0.46,1.66) *p* = 0.68	1.91 (0.82,4.45) *p* = 0.14
All-cause mortality	Female (187; 7, 76, 54, 24, 18 *)	1.51 (0.69,3.30) *p* = 0.30	1.00-reference	0.65 (0.45, 0.95) *p* = 0.0250	0.72 (0.44,1.18) *p* = 0.19	1.39 (0.78,2.48) *p* = 0.27
	Male (254; 9, 67,106, 39,18 *)	6.37 (3.15,12.89) *p* < 0.0001	1.00-reference	0.98 (0.71, 1.35) *p* = 0.90	0.83 (0.54,1.29) *p* = 0.42	1.79 (0.99,3.23) *p* = 0.05

* 8 deaths at females and 15 deaths at males with missing BMI.

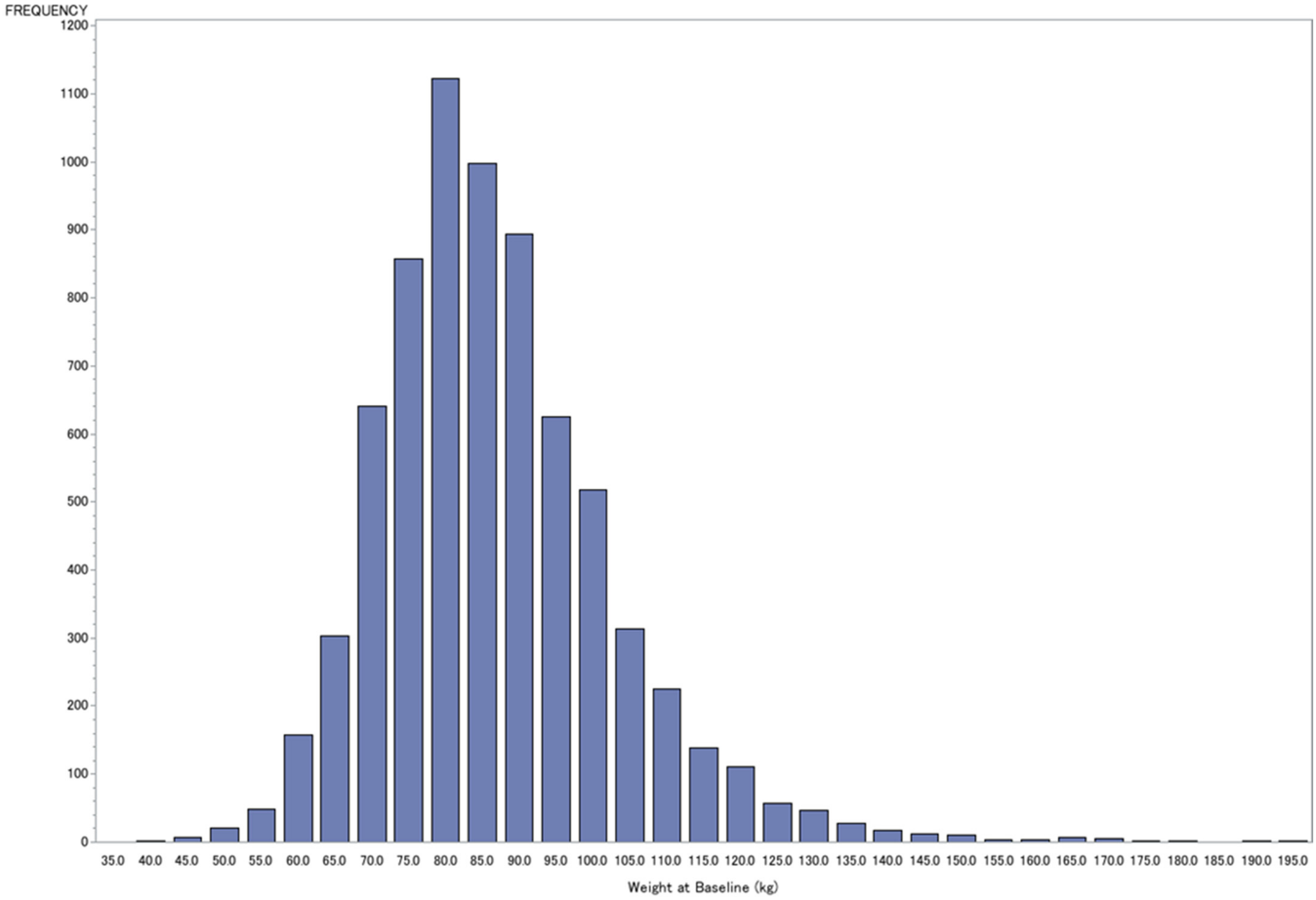

(**a**) Frequency males across the range of body weight at baseline

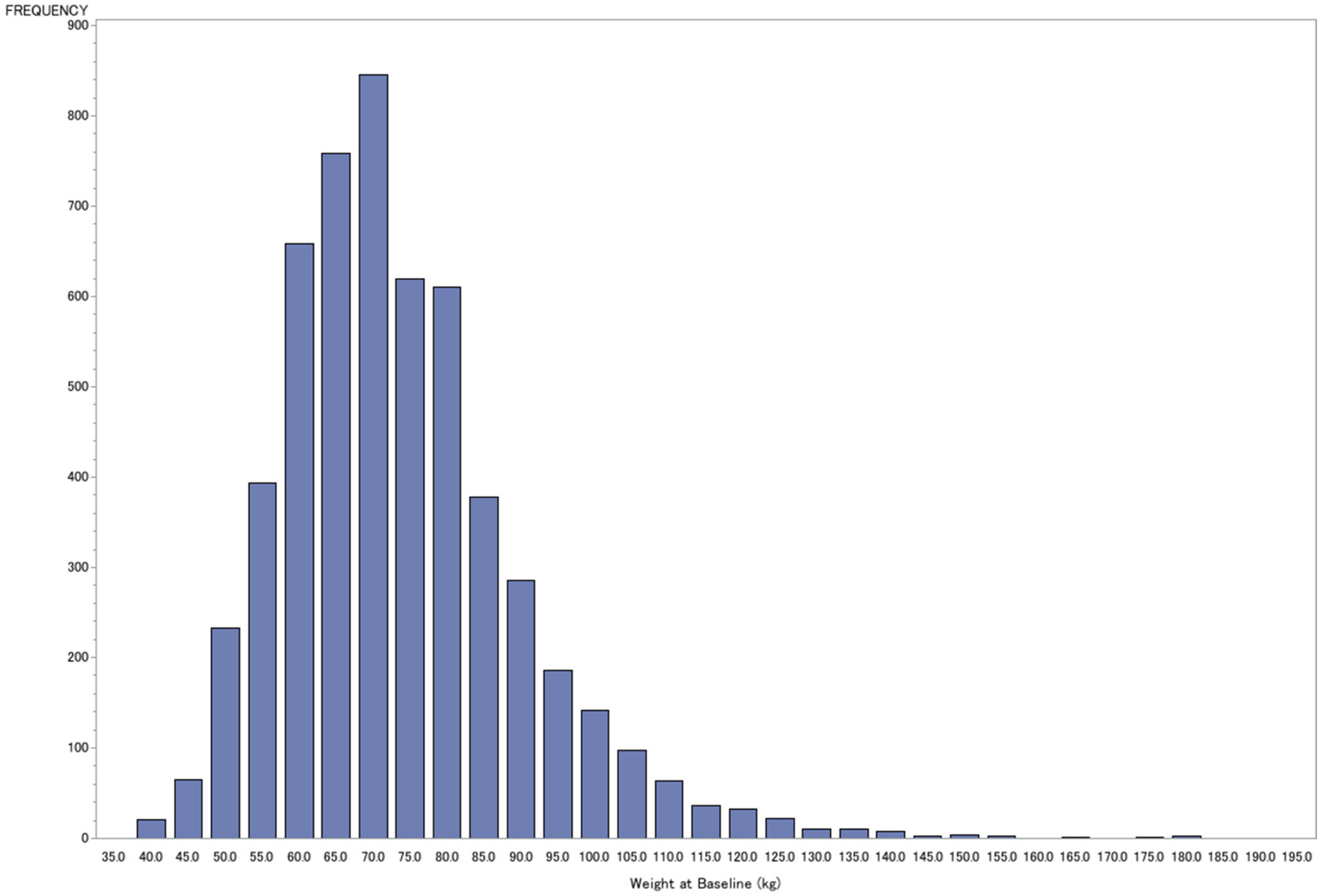

(**b**) Frequency females across the range of body weight at baseline

Figure S1. Frequency of patients (split by male and female) across the range of body weight at baseline. (**a**) Frequency males across the range of body weight at baseline, (**b**) Frequency females across the range of body weight at baseline.

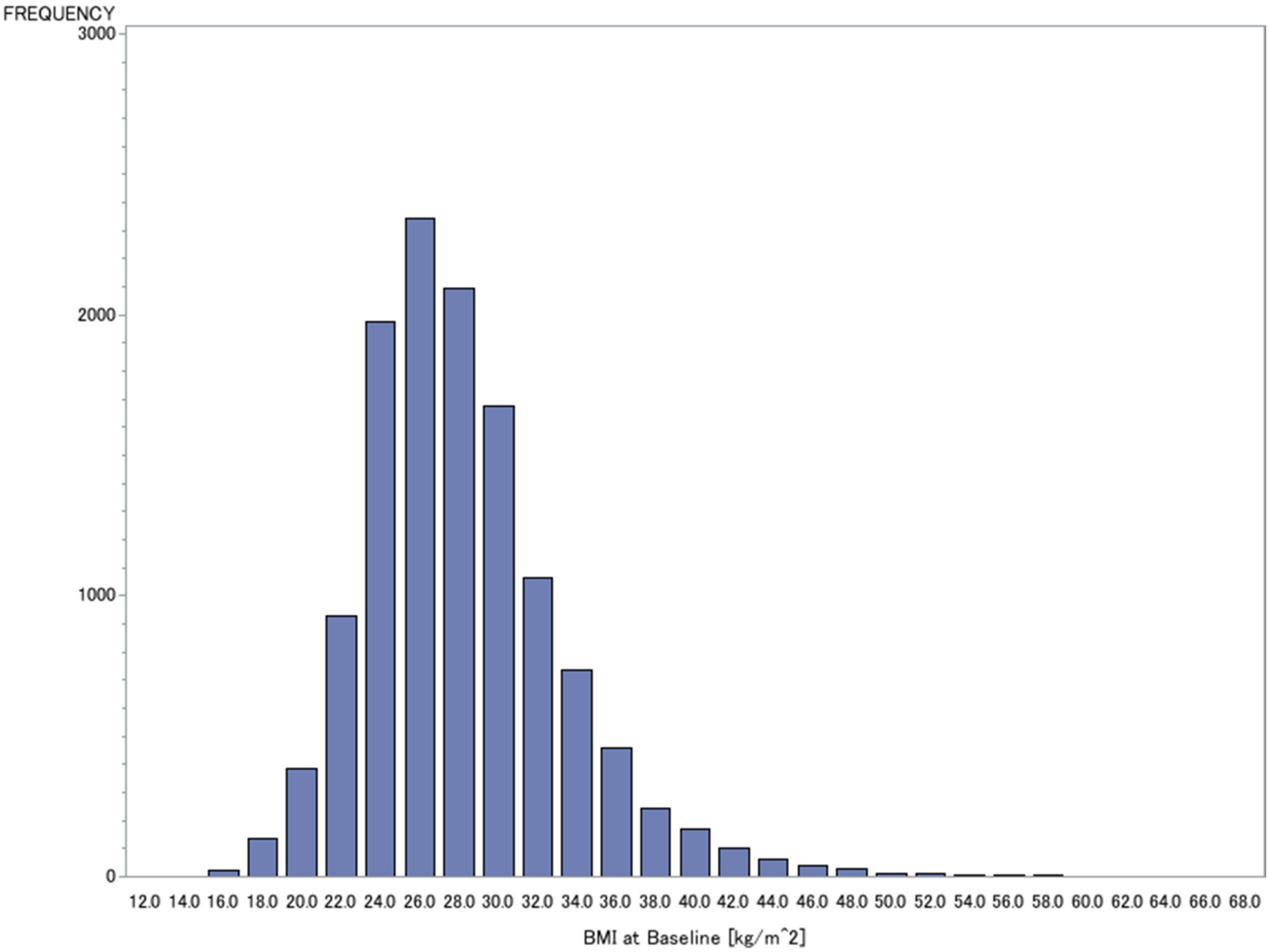

Figure S2. Frequency of patients across the range of BMI at baseline.

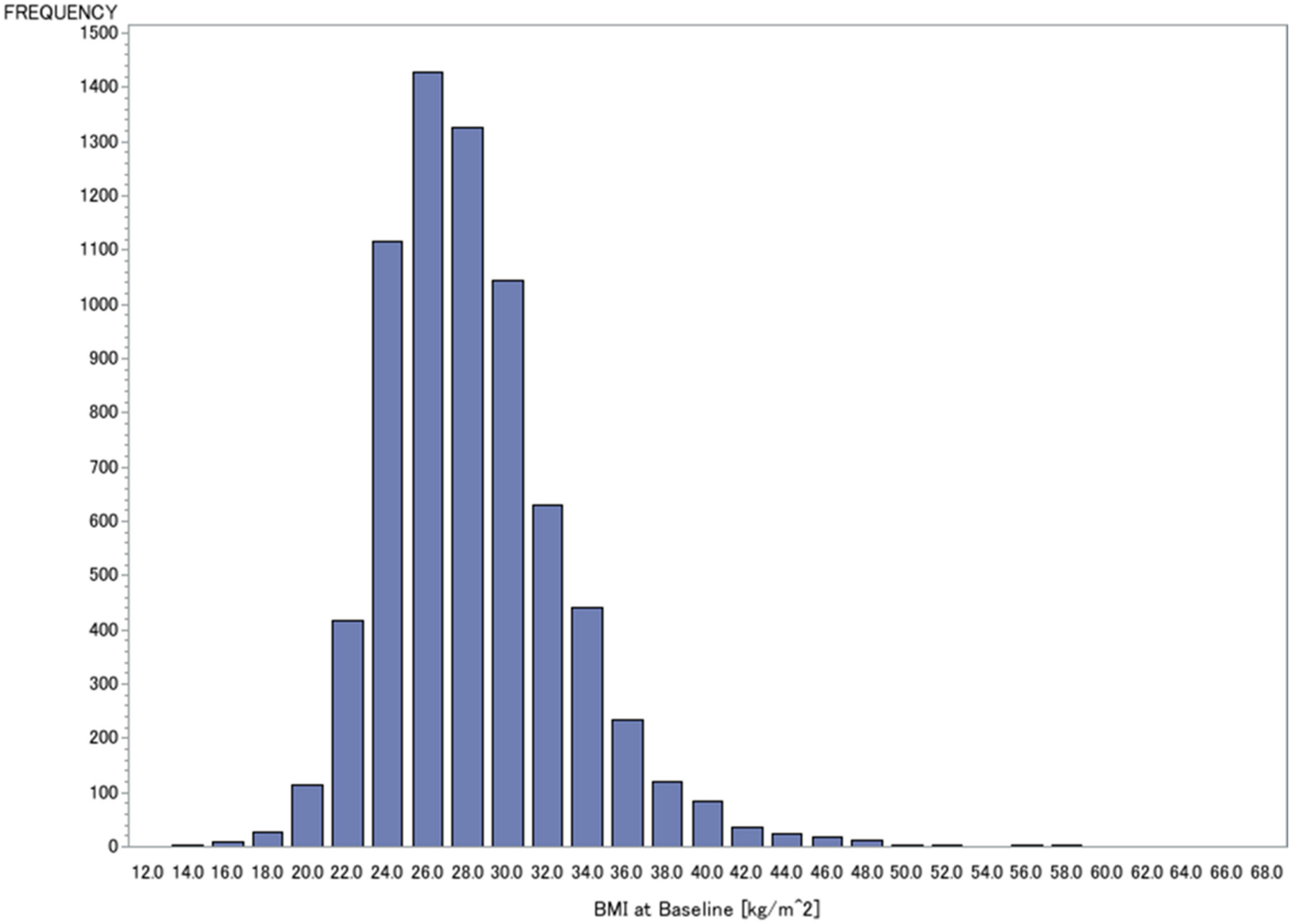

(**a**) Frequency males across the range of BMI at baseline

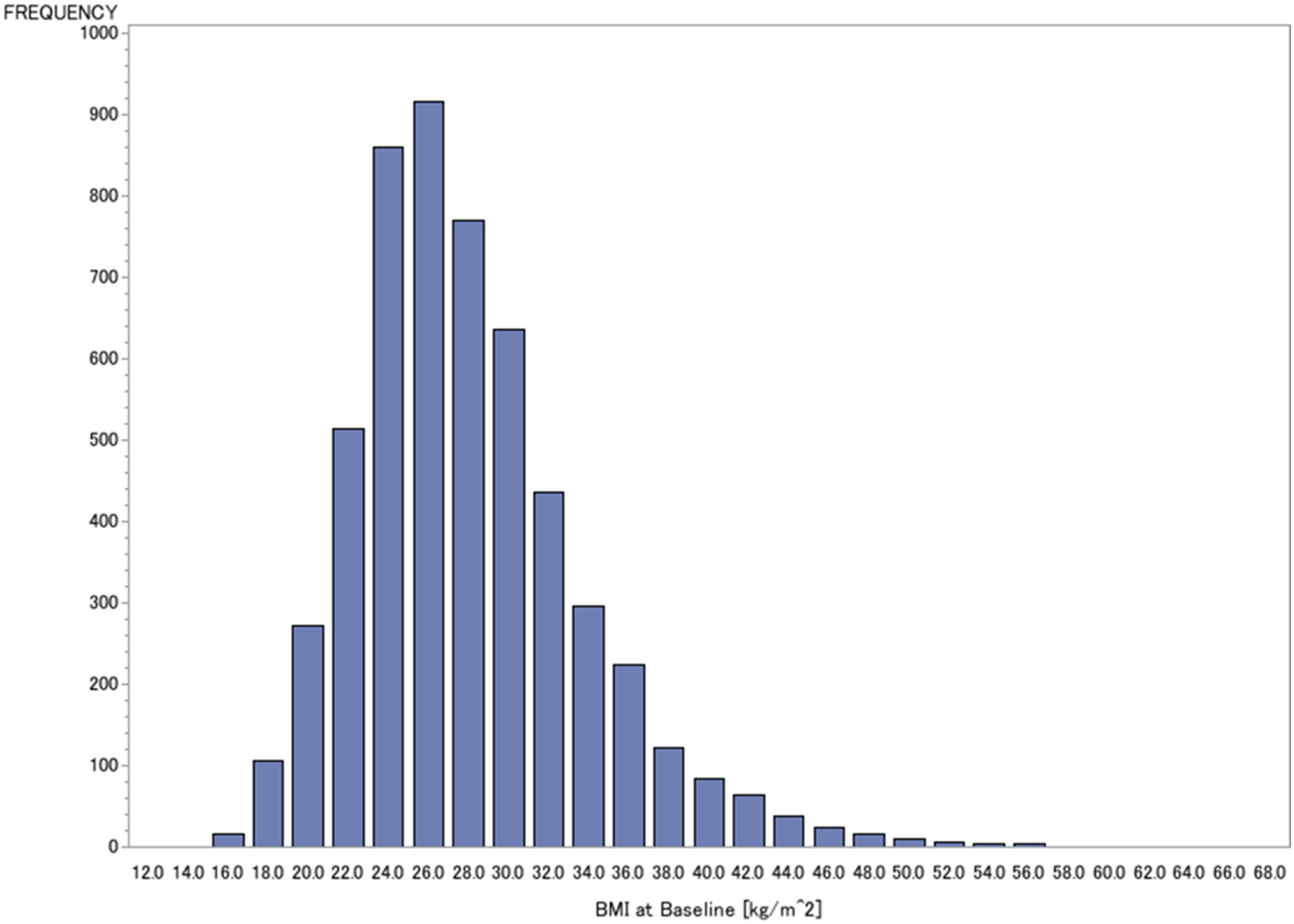

(**b**) Frequency females across the range of BMI at baseline

Figure S3. Frequency of patients (split by male and female) across the range of BMI at baseline. (a) Frequency males across the range of BMI at baseline, (b) Frequency females across the range of BMI at baseline.
